# Feeling existentially touched – A phenomenological notion of the well-being of elderly living in special housing accommodation from the perspective of care professionals

**DOI:** 10.3402/qhw.v8i0.20587

**Published:** 2013-04-18

**Authors:** Anette Lundin, Lars-Erik Berg, Ulla Hellström Muhli

**Affiliations:** 1 Schools of Technology and Society, Department of Social Psychology, University of Skövde, Skövde, Sweden; 2 Department of Sociology, Uppsala University, Uppsala, Sweden

**Keywords:** Well-being, phenomenology, elderly care, lifeworld, lived experience, existentially touched

## Abstract

This article presents a phenomenological analysis of interview material, in which 12 care professionals in elderly care reflect on the elderly's well-being within the frame of special housing accommodation. The perspective of the care professionals is of special interest. The findings show that the well-being is characterized as the elderly's *feelings of being existentially touched*. The well-being is an existential experience of being acknowledged as a human being and is an approach that classifies the elderly's needs as those of *having, loving*, and *being*. The meaning of the phenomenon is elucidated by the constituents: (1) *to feel the freedom of choice*, (2) *to feel pleasure*, and (3) *to feel closeness to someone or something*. The findings contribute new understanding of well-being in the elderly care by its existential dimension of the well-being as “just being” and of doing things in order to experience meaningfulness. Accordingly, the well-being of the elderly as it is seen from the perspective of the care professionals involves both carers’ subjectivity and intersubjectivity between the care professional and the elderly. An implication for promoting elderly's well-being is to develop awareness of these existential dimensions.

Approximately 8% of the Swedish population between the ages of 65 and 79 live in special housing accommodation (SHA) for the elderly. Among the very old people (80–89 years old), the amount of residents is 20%, and for the oldest segment (over 90 years), it is close to 50% (Whitaker, 2004). Considering that the number of those in the oldest range residing within SHAs has increased, the importance of studying the well-being of this population has also increased. The reason for this is that these groups of elderly have a greater need for social care and health services (Sherlund & Larsson in Dunér, 2007). They also have specific needs that affect many individuals. This category of elderly is then seen as incapable of managing on their own. With this, we imply that category cannot completely fend for themselves, owing to characteristics that put them at a disadvantage, e.g., very frail and those with mental or physical illness and disabilities. Thus, a value-based social and health care for this population is requested (SOU, 2008, p. 18). The value-based care is a range of standards aiming to improve the elderly's quality of life and well-being by ensuring that each person gets the care that is most appropriate for them as individuals. In the guidelines for a value-based care, words such as dignity, security, and well-being are used (SOSFS, 2012, p. 3). However, a proper description lacks on what the phenomenon of the well-being of the elderly living is at SHA.

Nevertheless, from the literature the following definitions can be retrieved: “Well-being is an emotional experience which does not require intact cognition” (Ericsson, 2012, p. 3). This definition suggests that, for example, elderly with dementia disease, living at SHA, and who are unable to communicate their needs are entitled to receive well-being supported care (Ericsson, 2012). Corinne (2005) describes the well-being as having a strong correlation to self-perception of health and states that the term *life satisfaction* has been used to describe well-being. Life satisfaction is connected to domains such as the person's financial circumstances, housing situation, main activity, and amount of leisure time. Satisfaction interfaces with needs and corresponds to the fulfilment of needs. Being pleased is thus an attribute of experience at a particular moment, while satisfaction refers to more inclusive domains of life. However, both refer to a state of being.

Referring to Corinne (2005), the loss of self-sufficiency leads to the specific inability to fulfil the needs. For that reason, it involves resorting to other persons for the accomplishment of these tasks. For example, an elderly person who loses his or her self-sufficiency might have to move to SHA in which care professionals would be able to help him or her daily in order to fulfil their needs for well-being. This posits some intriguing questions. Specifically, what is the care professional's experience of well-being of the elderly living at SHA? How do they understand the meaning of the phenomenon well-being? Therefore, our analysis focuses on well-being of the elderly as a phenomenon, understood in discursive terms that are presented by the care professionals.

## Well-being

With reference to Graham and Shier (2010), the well-being (often defined as subjective well-being) measure/scale originates in the social and caring sciences and sheds light upon how people make sense of their lives. This scale includes measure of satisfaction, lack of depression and anxiety, and positive moods and emotions. Within positive psychology, progress has been made in understanding the components of subjective well-being, the importance of adaptation and goals for feelings of well-being, the temperament underpinnings of subjective well-being, and its cultural influences. Representative selection of respondents, naturalistic experience sampling measures, and other methodological refinements are used to study subjective well-being (Diener, 2000). Well-being is also described from the social-contextual perspective which presents conditions that facilitate or forestall the natural processes of self-motivation and healthy psychological development. The findings have led to three innate psychological needs—competence, autonomy, and relatedness—which, when satisfied, yield enhanced self-motivation and mental health but, when thwarted, lead to diminished motivation and well-being (Ryan & Deci, 2000). According to Svensson, Mårtensson, and Hellström Muhli (2012), from the perspective of caring science, well-being entails both health and quality of life, whilst “being well” is one aspect of health which refers to the phenomenological concept of emotions and experiencing. Life can be valued from the degree of lust, reluctance, pain, and painlessness: a good life consists of feelings of pleasure, connotations of happiness, and satisfaction in a longer time perspective. However, well-being from the perspective of the elderly (women) themselves is emotional experiencing of continuity in the self-identity, and of sociality with others. This kind of emotional experience gives meaning to life *in situ* (ibid.).

An existential theory of well-being can be derived from the phenomenological notion of Heideggerian's existential philosophy of being-in-the-world (*Dasein*) (Swe. tillvaro) (Heidegger, 1927/1981). This perspective highlights the existential dimension of temporality, spatiality, embodiment, intersubjectivity, and mood as parameters from which the lifeworld of well-being could be understood as existential (Dahlberg, Todres, & Galvin, 2009). The being-in-the-world is an existence with other human beings as well as oneself (Sarvimäki, 2006). Thus, existence is characterized by a being-with (Dasein-with). Other persons are also Daseins: “the world of Daseins is thus in a very profound meaning a with-world /*…*/ In the with-world of Daseins, Dasein is defined as care”. (Ibid., p. 7)

This perspective also emphasizes authenticity—a self that seizes and defines itself—and a balance between existential dwelling and existential mobility. It suggests a balance between structure and process in the sense that our existence in the world needs both a foundation (familiarity) and a movement forward (evolvement). The mobility or the authentic movement forward could be exemplified as feelings of future possibilities connected to our life's desires. Well-being is then understood as all the ways in which we are able to have access to, and actualize a full range of experiential and behavioural possibilities (Todres & Galvin, 2010).

According to Todres and Galvin (2010), these possibilities are called *existentiale* by Heidegger, and include the above-mentioned spatiality, temporality, intersubjectivity, embodiment, and mood. Given this, well-being is related to both the *lifeworld* (Husserl, 1930/2004) and *everyday existence* (Heidegger, 1927/1981; Schutz, 1967, 1970) at the SHA. Accordingly, support for well-being is helping a person to experience existential mobility in all the ways he/she can (Todres & Galvin, 2010). However, existential mobility has to be balanced to existential dwelling, which means an existential homecoming that authentically grounds the human potentiality for peaceful attunement to existence (ibid.). The dimension of well-being is then understood as being-in-the-world of acceptance and the possibility of peace. With that, in this study, the *lifeworld perspective* of well-being as dwelling—mobility assumes to be analysed from the basis of the care professionals’ experiences of *meaning-making*, and understanding of the phenomenon of well-being in order to be able to support for well-being of the elderly at SHA. How the care professionals then support for well-being is the subject of a next article.

The term *care professional* is used in order to emphasize that the personnel as carer at SHAs for elderly have a commission and are expected to provide care within the frames of political, institutional, and organizational values and goals. Hence, the term *carer* will be used to indicate care professionals.

## Methods

### Design

The design of this study draws upon a phenomenological tradition of description (Dahlberg, Dahlberg, & Nyström, 2008), and the analysis of the data material about well-being is related to the key concepts of *lifeworld* and *meaning-making*. The notion of the lifeworld is central in phenomenology and means the concrete and lived existence in the world. Still it is often disregarded (Dahlberg et al., 2008). Accordingly, the phenomenological attitude to science and research means to describe the world in the way that it is experienced by humans, what the world is and means to humans, and how humans relate to this world, to each other, to different situations—to all possible “things” of the world (ibid, p. 6). The reason for this description is that human life manifests itself in experience.

As shown, the design and carrying out of the research is based on a reflective lifeworld approach (Dahlberg, 2006). This approach claims a sensitive openness and attitude to the human, using interview methodology (Kvale, 1997). In order to understand the carer's experiences from the interviews, the analysis includes Heidegger's concept of *existence* and *being-in-the-world* (Dasein) and *meaning* to describe the human ordinary everydayness (Heidegger, 1971). This phenomenological knowledge not only brings about an understanding of the natural attitude to things, how things appear for someone, but also in a second analytical step, the way that the things are experienced as phenomena (Dahlberg et al., 2008). As regularities in experiences are theorized, it is possible to become aware of the character and conditions of the phenomenon of well-being. This analysis aims to shift the natural attitude of the phenomena to the phenomenological scientific attitude. The phenomenon is described to be a structure of essential meaning that explicates a phenomenon of interest, here the well-being. Thus, we seek the patterns of meanings of experience, and we want to grasp the meaning of the phenomena in focus (well-being), which are analysed, synthesized, and presented in the “Results” section.

Phenomenology starts with practice, in this case, the carer's experiences of well-being in the care practice. It theorizes the universalities of the practice, and returns the knowledge to its practical context. This research process sets the researchers as participants in the relationship between themselves and the world that they experience and want to describe, an opposite position from that of traditional positivistic research. Accordingly, researchers place themselves in the position of contributors to the meaning of the world and, therefore, have to be aware of their contribution as descriptions of the meaning. The reflective lifeworld approach makes this relationship of particular importance (Dahlberg et al., 2008).

Dahlberg (2006) uses the term *bridling* which concerns restraining the researcher's pre-understanding (in the form of personal beliefs, theories, and other assumptions that would mislead understanding) and thus facilitating openness. Bridling is about “paying attention to how phenomena and their meanings are made explicit” (Dahlberg et al., 2008). Therefore, bridling is the suggested phenomenological attitude that encourages active waiting for the phenomenon to show itself to the researcher.

In our reports about the carers’ notions of the phenomenon of well-being of the elderly, we must describe both the whole structure and its constituents (which bring the flavour), and we must start with the phenomenon and then move on to the constituents (so that we know what they are constituents of). Everything is experienced as *something*.

### Procedure

Interviews with carers were conducted in May 2011 in two wards at a SHA in Western Sweden. This SHA was chosen due to accessibility and willingness to participate. At a meeting between the organization and the research group, representatives for the carers were informed about the design of the study. The ethical considerations used in this study builds upon notions of what must be reconsidered when conducting qualitative research: the autonomy, beneficence, non-malfeasance, and justice—as regulated in the Declaration of Helsinki (WMA, 2008). The participants were informed about the study as well as the issue of privacy, that participants’ identities would not be revealed and that participation was voluntary. The Regional Ethical Review Board, University of Gothenburg, Sweden, approved the proposal for this study on 21 March 2011 (reference nr: 669–10).

### Participants

In total, 12 interviews of an open kind (Creswell, 1998; Kvale, 1997) were conducted. The participants/informants were 12 women who have worked in elderly care for 10–34 years. They have worked at the studied SHA for 2–25 years. They were assumed to contribute data with rich variation, as they have different length of working experiences and are of different age and generations. The informants are called number 1 to 12 (I1–I12) in the Results section. When presented, the results will quote the participants using a written language, rather than a spoken language, in order to make the participants more anonymous. It will also help with the English presentation of the quotations. When a Swedish phrase or word does not have a direct translation into English, the Swedish phrase or word will be presented, using hard parentheses.

### Data collection

The first author conducted the interviews as open dialogues with a focus on *what* and *in what way* the interviewee experienced the phenomenon in question. The interviews varied between 25 and 50 min. The interviews were recorded on an MP3 player and began with an open-ended question: “What is well-being?” The interviews were conducted as dialogues in which the individual carers could tell their experiences as a dynamic co-production between the participant and the researcher. During the interviews, follow-up questions such as: “How did you experience that?” and “Can you give an example” were asked in order to gain an in-depth understanding of the phenomenon and to understand what well-being meant for the specific carers. The interviews were transcribed verbatim (Linell, 1994).

### Data analysis

The analysis can be described as a movement between whole—parts—whole with inspiration from Dahlberg et al. (2008). The verbatim transcripts were read several times to get a feeling for each interview as a whole. This feeling consisted of an intuitive understanding of larger patterns and was supposed to be a guide to objective detail analysis. Thereafter, we found patterns of main constituents in each interview as well as between the interviews. Through this analysis of patterns, we found the phenomenon: *well-being for elderly living at SHA is about feelings of being existentially touched*. The analysis has been characterized by movement between the intuitive whole and the person-related patterns, and further to meaning units and their cores. The work has been reflective and critical, and has been characterized by re-readings in order to allow the empirical material to be confirmed. When doing these re-readings, bridling (Dahlberg, 2006) became of high importance as a way of not letting preconceptions misguide the finding of a pattern.

## Results

The phenomenon well-being of elderly at SHA (the what), experienced by the carers, are characterized as the elderly's feelings of being existentially touched. In other words, well-being is an existential experience of being acknowledged as a human being, and is an approach that classifies the elderly's needs as those of *having*, *loving*, and *being*. The *having* relates to have or not have the abilities to fend for themselves, *loving* relates to feel loved or needed, and *being* relates to the state of being or not being left existentially alone. The carers relate to these feelings from the caring position by pointing out the phenomenon of well-being as substantially existential, however, as different from their own way of experiencing well-being as it is here exemplified by I6:

Just sit and watch your life go by and watch people passing/*…*/Because if nobody sees you or talks to you as everyone just runs by … then you do not exist. Or, I would not feel as if I was somebody. If I was in a wheelchair and everyone passed me by without saying a couple of words, stroking me or touching me or even seeing that I was sitting there … It would be awful, horrible. It would be the worst lack of well-being.

This existential touch refers to well-being regardless of the specific inability that is the reason to moving to and to the life at SHA. The phenomenon well-being as elderly's feelings of being existentially touched is manifested in the data material in the shape of varying constituents. Those are: (1) *to feel the freedom of choice*, (2) *to feel pleasure*, and (3) *to feel closeness to someone or something*.

### To feel the freedom of choice

The first constituent is *freedom of choice*. It elucidates the meaning of *being existentially touched*. This is a highly valued aspect of well-being. Freedom means that the elderly person is satisfied when having *the freedom of choice* and ability to choose. Ability to choose is also applied to sleeping or to freedom from being put to bed against one's will. Ability to choose is also about taking part in activities, sometimes in order to avoid boredom as I2 puts it: “They are in need of activation, or freedom from activities they do not want to perform and further, freedom to choose mealtimes”. This ability to choose is also stressed as an aspect of the enjoyment and is exemplified as visiting other places (*mainly a nearby supermarket*) and buying clothes or a treat to go with your coffee. This enjoyment is contrasted with the *limited abilities to choose* at the ward: “Here you get the coffee that is served and cannot choose your Danish by yourself”. (I1)

Well-being as a feeling of freedom to choose is also about the daily routines. Routines give recognition and provide the possibility not to choose. Routines are described as being especially important when the particular resident has a further progressed dementia and does not have the ability to choose. In these cases especially, well-being is achieved “when everything moves at its ordinary pace” (I5). However, routines vary from resident to resident depending on their needs, and that is something that is needed to be learned to manage, as I5 describes it:

You learn it [to manage different needs] gradually. That ‘we do it like this, then it will be fine’, ‘no, now we did it like this and it went totally wrong last evening’, ‘yes, she gets to sleep until she awakes and yes, that was very well’ or ‘you are not allowed to awake her in the mornings because then it will be chaos’. You get to learn all of them, all of them are different. It can be such a simple thing that you do not know and you let her eat with a fork instead, because you do, but she usually eats with a spoon. Then she might not eat, and then she might not eat all day. It is such a small thing that triggers a lot. And it gets so wrong.

The carers point out the importance of the acknowledgement of the needs for routines, as the residents’ well-being, particularly when the residents cannot speak for themselves.

### To feel pleasure

The next constituent *feeling pleasure* elucidates the meaning of the phenomenon of *being existentially touched, by* showing a state of acceptance, and peaceful unison to existence. The pleasure relates to “just being” and is exemplified as the enjoyment of sitting out in the sun or lying in bed or not wanting to be activated, as I4 expresses it: “Maybe many may not want that much to do”. The elderly are offered many activities at the connected activity centre and one of them is musical entertainment. One accordion player, in particular, is described as a favourite among the elderly residents as listening to him brings about recognition—both for one of the residents who has played the accordion himself as well as for the others who recognize old tunes. I7 uses the word “tearful” to describe the expression of feelings among the residents who participate in this activity and she states: “then [when the accordion player comes for a visit] it is well-being for many”.

Well-being is also feeling of pleasure when meeting a familiar face and that the carers “feel” that the elderly residents are recognized, as well as having time for the individual elderly, as it is expressed by I8: “that they feel that we have time for them”. However, to elucidate the meaning of this phenomenon, the carers contrast the elderly's needs as well as their well-being with the carers themselves. I1 notes that “well-being is not the same for us as for them. II have higher demands”. I4 points out the different perspectives by saying that “some can feel fine by lying in their bed even if we do not think so, we might think that they should get up and sit and such”.

The feeling of pleasure is likewise described by accounting the opposite positions as: not being able to go to the bathroom when you need to, not being allowed to sleep-in in the mornings, not being changed, not getting good food, and no help in getting up, not getting your feet taken care of, not getting your nails done, and not getting your hair done if you are used to that. As a summary of what it is to feel pleasure, and what gives this pleasure, I1 and I8 express: “it is the little things”.

### To feel closeness to someone or something

The third constituent, *closeness to someone or something*, elucidates the meaning of the phenomenon of *being existentially touched*, by the closeness, for example to the carer such as sitting down to make the residents feel closeness. A social dimension to the primary needs is pointed out as the importance of feeling a sense of belonging to someone: a resident, a carer, or a relative. I11 says: “It can be that they need a hug, that they need close contact”. She does not know why it helps; just that it does, and wonders if it can be “that they feel that you care about them”. Closeness elucidates the meaning of the phenomenon *as* being with others, and it is expressed as the most important factor for well-being. I10 describes how she tries to finish her work in order to sit down with one of the residents to watch a game show, and how it is sitting next to the resident for that half an hour, which makes the resident happy.

Well-being is also about feeling closeness to places and the former home and even to the SHA that is the last home of the elderly. However, for some, the change in the elderly's everyday existence, due to moving into SHA and/or having diseases, cause anxiety. I3 expresses this as follows:

Nothing will become like it was before in life when you end up in a residency… It actually is life's last halt many times or always. Often they have had a stroke or might be put in a wheelchair and have become paralyzed, lost speech or sight. It is so various what has happened. Or, dementia … It is a lot that affects well-being.

Closeness by sitting down and talking seems to give well-being. This is true even when it concerns anxiety followed by the dementia disease. I12 puts it as follows: “you sit down and try to talk as calmly as possible and explain”.

## Discussion

The contribution of this article will be discussed in relation to substantive, theoretical, methodological, and general practical implications. The result about the existential dimensions of well-being is highlighted as a new understanding of well-being of elderly at SHA in a Swedish elderly care setting.

### Substantive implications

The analysis contributed to an understanding of how the carers of an SHA understand the well-being of the elderly residing at an SHA. The experiences of well-being turned out to consist of the elderly's feelings of being existentially touched. This essence showed itself in the constituents of (1) “feeling freedom of choice”, (2) “feeling pleasure”, and (3) “feeling closeness to someone or something”.

1. The experiences of the importance of elderly's *feelings of freedom of choice* when it comes to their well-being, show an understanding of different needs when it comes to being activated, having routines and having a choice. This recognition shows the importance of existential mobility (Todres & Galvin, 2010). The elderly need access to actualizing experiential possibilities.
2. The experiences of the importance of the elderly's feeling of pleasure when it comes to their well-being, shows an understanding for different needs when it comes to “just being”. For example, the perception that being activated is good for human well-being is widespread and supreme for carers. However, the results of this study show that well-being may also involve the inactivity of “just being”. Thus, while being activated is one way of being authentic and being-in-the-world (Heidegger, 1827/1981), “just being” is another. This notion of just being shows the carers’ recognition of the need of dwelling (Todres & Galvin, 2010) for the well-being of elderly. The elderly living at SHA is in need of familiarity and a sense of belonging in their everyday existence.
3. The experiences of the importance of the elderly's feelings of closeness to someone or something when it comes to their well-being, shows an understanding of *“just being with”* as important for the well-being. It shows recognition of the importance of a more elementary form of interaction between the carers and the elderly. It also shows the importance of creating a shared existence at the SHA in order to support the well-being of the elderly residing there. The lifeworld of the elderly at the SHA is a *with-world* (Sarvimäki, 2006, p. 7) shared with the carers and the other residents.

The main empirical questions of this study were “What is the care professional's experience of well-being of the elderly living at SHA? How do they understand the meaning of the phenomenon well-being?” These questions were answered through this phenomenological approach through the descriptions of the experiences given by the carers. The answers to these questions were found in the general construct of the phenomenon of elderly's well-being as consisting of feelings of being existentially touched.

### Theoretical implications

Praxis in elderly care has been to decide on well-being from external and medical criteria, the assumption being that experts can decide what well-being is for elderly. They are objectified to speak in a friendly way, with the intention that they will feel good. However, the phenomenon of well-being as feelings of *being existentially touched* and its constituents of: (1) *to feel the freedom of choice*, (2) *to feel pleasure*, and (3) *to feel closeness to someone or something*, entail a notion of the phenomenological Heideggerian existential philosophy of being-in-the-world (*Dasein*). This indicates the necessity to meet the elderly's subjectivity. Such a notion is in line with the conceptualization by Caldas and Berterö (2007). They emphasize that existential care consists of the carer understanding the subjective world of the care recipient and experiencing union with him or her (p. 381). In this mutuality, care build upon the meaning making of the care recipient.

Subsequently, the question to be asked here is: what does it mean to feel being existentially touched? The concepts of authenticity, dwelling, and mobility (Dahlberg et al., 2009; Heidegger, 1927/1981) contribute to a fuller understanding of the well-being as a mode of existential being as “just being”. Accordingly, we also understand that “just being” in everyday life or “just being” with the elderly and doing things, gives well-being in order to experience meaningfulness. The understanding of well-being as existential in an elderly care context can be answered and organized as the elderly's needs as those of *having, loving*, and *being*. Through this phenomenological framing, a broader understanding of the phenomena (the essence) has, thus, been explicated. This framing is working as a background against which the phenomenon of well-being stands out as a structure of meanings, as it has been understood by the carers as well as by the researchers. Thus, the meaning of the well-being belongs to the “lifeworld” and illuminates the essential characteristics of the phenomenon well-being as *feelings of being existentially touched*. In this way, we can say that our findings from this phenomenological analysis include a general meaning as well as the individually perceived and lived meanings of the carer about elderly's well-being.

### Methodological implications

A phenomenological approach was chosen in order to present universalities which can lay a foundation for discussing needs or changes when it comes to professional caring in the setting of municipal elderly care at SHAs. This study was inspired by the concept *bridling* (Dahlberg, 2006; Dahlberg et al., 2008) as it embraces an awareness of our natural attitudes and promotes an openness by taking a more reflective position. The bridling in this study has been expressed through an inductive analysis of empirical findings, showing us what needs to be understood theoretically. The bridling also took place when finding the essence and its constituents, as these were patiently waited for and revisited in the empirical material. The bridling enabled us to let the voices of the carers show what the well-being of the elderly living is at SHA.

In lifeworld research, “the idea of generalization must be problematized” (Dahlberg et al., 2008). Our study has produced knowledge about carers’ understanding of elderly's well-being when living at SHA, but it has also gone beyond that limit through presenting the phenomenological results as always being contextual (ibid., p. 343) but are lifted from their concreteness through the general structure of the phenomenon and its constituents. By studying the whole and the parts as interrelated, it has been possible to obtain an understanding of their relationships and the context in which they arise. It is through application that usefulness and the possibility to use the findings in different contexts can be evaluated.

### General practical implications

In order to promote the well-being of elderly living at SHA, an awareness of the existential dimensions must be developed. The carers and the elderly live in a shared existence, an intersubjective world, as well as being subjects. It is of importance that these dimensions are recognized as there is a risk that the relationship objectifies the elderly as he or she is reduced to primary needs which are taken care of within the resources available if this intersubjectivity does not exist. There is a need for time reserved for just *being with* the elderly and *being able* to support their needs for individuality through going for walks, taking them out for coffee, or taking care of their hair. The allocation of resources should take this quality of care into consideration.
